# Multi-Layer Biocarbon Carbonized from Cellulose Nanocrystals as a Novel Lubricant Nanoadditive in Rapeseed Oil

**DOI:** 10.3390/ma19081483

**Published:** 2026-04-08

**Authors:** Minghang Guan, Kaiqi Su, Guodong Chen, Yu Cheng, Chao Chen, Haibin Zhou, Xiubo Liu, Yuan Meng

**Affiliations:** 1 School of Materials Science and Energy, Central South University of Forestry and Technology, Changsha 410000, China; 13127733797@163.com (M.G.);; 2 State Key Laboratory of Powder Metallurgy, Central South University, Changsha 410083, China; 3 College of Materials Science and Engineering, Taiyuan University of Technology, Taiyuan 030024, China; 4 Hunan Province Key Laboratory of Materials Surface & Interface Science and Technology, Changsha 410000, China

**Keywords:** biomass derivative, lubricating additive, friction reducing, antiwear, lubrication mechanism

## Abstract

It is limited to use cellulose nanocrystals (CNCs) as green lubricant nanoadditives due to their high biodegradability. A promising solution is to convert CNCs into biocarbon. Herein, a multi-layer biocarbon (MLC) was prepared by carbonizing CNCs with an ionic liquids–thermal method. MLC was characterized comprehensively and then dispersed into rapeseed oil for use as a nanoadditive. The tribological performance of the MLC nanoadditive was evaluated using a ball-on-disc tribometer. The lubrication mechanism of the MLC nanoadditive was elucidated according to wear analysis of the worn surfaces and wear residues. It was found that MLC had a high carbon content of 77 at% and showed a two-dimensional multi-layered morphology. Each layer was composed of amorphous carbon nanosheets embedded with many crystalline carbon dots. The MLC nanoadditive was of excellent dispersibility and stability in rapeseed oil. Tribological experiments showed that the MLC nanoadditive, with a concentration of merely 0.04 wt%, led to a decrease in the frictional coefficient by 12.4% and the wear volume by 50.7%, having higher efficacy than the CNC nanoadditive. The exceptional lubrication effect of the MLC nanoadditive was mainly attributable to its interfacial deposition behavior and its subsequent fragmenting behavior. This work develops a novel method for biocarbon preparation and showcases its significant potential in lubrication applications.

## 1. Introduction

Cellulose nanocrystals (CNCs), a typical biomass nanomaterial, has increasingly attracted attention in the field of lubrication due to its unique advantages [1,2,3,4], such as a low density, high crystallinity, high mechanical strength, greenness, and renewability. It has been reported that CNC shows great potential for use as a new type of sustainable lubricant nanoadditive for reducing friction and wear. On the one hand, the CNC is inborn with high suitability for use as water-based lubricant nanoadditives [5,6,7,8], because its surface is rich in plenty of hydrophilic hydroxyls, thereby leading to its superior dispersibility in water-based systems. As an example, Zakani et al. [5] reported that a CNC aqueous solution, in a mass percentage of ca. 1 wt%, resulted in a 25% decrease in the frictional coefficient and a 30% reduction in the wear rate. On the other hand, the CNC is incompatible with low-polar media also due to its surface hydroxyls, which is a major barrier to using it as an oil-based nanoadditive. Accordingly, the CNC is usually needed to increase its lipophilicity via appropriate surface modifications, including acylation [9,10], esterification [11,12], etherification [13,14], silanization [15,16], sulfonation [17,18], etc., which commonly take the hydroxyls as the reactive site. For instance, Li et al. [9] found that CNC grafted by stearoyl chains was well dispersed in PAO oil and it reduced the frictional coefficient by 30% and largely smoothed the worn surface through a mending effect. Even though it is quite promising to use CNCs as nanoadditives, its limitation should be noted because CNCs are readily biodegradable [19]. It is hard for CNCs to not deteriorate for long in various liquids, especially aqueous media, e.g., a CNC aqueous solution can only be stored for ca. 6 months even under low-temperature conditions.

Carbonization treatment is deemed a promising solution to the above limitation. During a typical carbonization process, hydrogen and oxygen elements are greatly decreased, whereas the carbon element is largely enriched, which will effectively enhance the resistance of biomass to biodegradation. In addition, considering that the CNC can be converted into a new-type biocarbon after appropriate carbonization treatment, we hypothesize that this biocarbon should be nanosized and it probably has excellent tribological performance just like some typical carbon nanomaterials (e.g., carbon dot [20], carbon nanotube [21], and graphene [22]). In general, there are two approaches for carbonizing biomass, i.e., high-temperature carbonization and hydrothermal carbonization [23,24,25,26]. The former is performed at a high temperature above hundreds of degrees centigrade accompanied by a vacuum condition or inert atmosphere, which inevitably results in high energy consumption and stringent equipment requirements. The latter uses water as the reaction solvent or medium and is carried out in a sealed pressure vessel at the temperature between 150 and 375 °C. Even though the reaction temperature for hydrothermal carbonization is seemingly lower as compared to high-temperature carbonization, a high demand for the facility is still needed due to its high pressure. Accordingly, it is challengeable to carbonize CNC at a relatively low temperature and atmospheric pressure.

Ionic liquids (ILs), a green solvent with noninflammability, high thermal stability, recyclability, and environmental friendliness, have been widely used for preparing and modifying nanomaterials [27,28,29,30,31]. As documented in the literature, CNCs can easily dissolve in appropriate ILs to form a homogeneous dispersion [32,33]. Thus, based on hydrothermal carbonization, it is quite likely to carbonize CNCs using a solvothermal method in which the water solvent is replaced with ILs. This improvement also comes with an additional advantage that no pressurization is needed for the IL-thermal carbonization method due to the extremely low vapor pressure of ILs. As a result, it is enough for the IL-thermal method to use some common glassware, which will greatly simplify the apparatus. In addition, ILs are generally catalytic [34,35], which can help lower the temperature for carbonizing CNCs. However, it has not been reported yet to carbonize CNCs into biocarbon using the IL-thermal carbonization method.

In this work, a novel multi-layer biocarbon (MLC) was prepared via an IL-thermal method using CNC as a precursor at normal pressure. MLC was characterized with X-ray diffraction (XRD), Fourier transform infrared spectroscopy (FT-IR), Raman spectroscopy (RS), Thermogravimetric analysis and Derivative thermogravimetry (TGA & DTG), X-ray photoelectron spectroscopy (XPS), and transmission electron microscopy (TEM). Moreover, MLC was applied as a lubricant nanoadditive in rapeseed oil. Its friction-reduction and antiwear performance was thoroughly evaluated on a ball-on-disk tribometer. The resultant worn surfaces and MLC wear residues were analyzed with scanning electron microscopy (SEM), white light interferometer (WLI), RS, and TEM. Also, the possible lubrication mechanism was elucidated for MLC nanoadditive.

## 2. Materials and Methods

### 2.1. Materials

CNCs, with >99% purity and ca. 1.45 mmol/g carboxyl groups, were purchased from Tianjin Woodelf Biotechnology Co., Ltd., Tianjin, China. The ionic liquid, 1-ethyl-3-methylimidazolium hydrogen sulfate ([EMIM]HSO_4_), was provided by Shanghai Macklin Biochemical Co., Ltd., Shanghai, China. Anhydrous ethanol, with 99.9% purity, was procured from Tianjin Fuyu Fine Chemical Co., Ltd., Tianjin, China. Petroleum ether, with 99% purity, was acquired from Sinopharm Group Co., Ltd., Shanghai, China. Refined rapeseed oil, selected as a base oil, was obtained from Wako Pure Chemical Industries Co., Ltd., Osaka, Japan. GCr15 steel balls with 6 mm sphere diameter, 62–66 HRC hardness, and G10 surface accuracy were purchased from Shandong Huamin Steel Ball Joint-stock Co., Ltd., Jinan City, China. GCr15 steel discs (36 mm diameter × 3 mm thickness), polished in our lab to achieve ca. 50 nm surface roughness, were supplied by Jiangsu Maote Steel Co., Ltd., Huaian, China.

### 2.2. MLC and Its Nano-Oil Preparation

MLC was prepared following an IL-thermal carbonization method, as described below. Freeze-dried CNC was ground into a fine powder. Next, CNC powder and [EMIM]HSO_4_ were mixed at a mass ratio of 1:50 under mechanical stirring to obtain a homogeneous mixture. The mixture was heated to 150 °C under Ar atmosphere with magnetic stirring for 3 h and then naturally cooled to room temperature. The resultant product was collected through centrifugal washing with ethanol 4 times. The product was dried under a vacuum at 80 °C for 4 h and designated as MLC.

The nano-oil was prepared according to a so-called solvent exchange method [36,37]. Specifically, MLC (or CNC) dry powder was homogeneously dispersed into ethanol with ultrasonic treatment operating at 300 W for 30 min, and then its wetted precipitate was collected via high-speed centrifugal separation for 2 min. The ethanol-wetted MLC (or CNC) was added into rapeseed oil and then subjected to ultrasonic oscillation at 400 W for 30 min. Accordingly, the nano-oils of rapeseed oil with MLC (or CNC) at a series of mass fractions (0.005, 0.01, 0.02, 0.04, 0.08 wt%) were prepared following the above procedure.

### 2.3. MLC and Its Nano-Oil Characterization

XRD measurements were performed with a Bruker D8 Advance X-ray diffractometer operating at 40 kV and 40 mA using Cu–Kα radiation. FT-IR spectra were recorded in transmission mode with a Thermo Scientific Nicolet iS20 fourier transform infrared spectrometer (Thermo Fisher Scientific, Waltham, MA, USA). Raman spectra were obtained using a 633 nm laser on a Thermo Scientific DXR3 smart Raman spectrometer (Thermo Fisher Scientific, Waltham, MA, USA). TGA and DTG measurements were performed on a Netzsch TG 209 F3 Tarsus analyzer (NETZSCH-Gerätebau GmbH, Selb, Germany) under N2 gas flow at a 10 °C/min heating rate. XPS spectra were recorded on a Thermo Scientific EscaLab 250Xi X-ray photoelectron spectroscope (Thermo Fisher Scientific, Waltham, MA, USA) using Al–Kα radiation and a passing energy of 20 eV. TEM imaging was performed on a JEOL JEM-F200 microscope operating at 200 kV.

The nano-oil was imaged with a Sony IMX789 camera (Sony Corporation, Tokyo, Japan) after standing for several days (1, 7, and 30 days) and then analyzed for particle size distribution using a Malvern Zetasizer Nano ZS90 instrument (Malvern Instruments, Malvern, UK) to assess the dispersion stability.

### 2.4. Tribological Experiment

The nano-oils were evaluated for tribological behaviors on a BMXW-002 ball-on-disc tribometer (Jinan Boyan Huachuang Intelligent Technology Co., Ltd., Jinan, China). The tribopair, comprising a steel ball (upper) and a piece of steel disc (lower), was always flooded in an oil bath during the tribotest. In the ball-on-disc configuration, the disc rotated against the stationary ball with a 5 mm contact radius. The tribological experiment was conducted at an ambient temperature of 24 ± 1 °C for 30 min, and a series of normal loads (7.5, 10, 12.5 and 15 N) and rotational speeds (100, 150, 200 and 250 rpm) were applied. The steel ball and disc were subjected to 5 min ultrasonic cleaning in petroleum ether before each experiment, with subsequent air drying. The experiment was performed three times or more under each experimental condition to guarantee repeatability. The frictional coefficient was automatically acquired through the computer system of the tribometer. The wear volume measurements were conducted on a profilometer (MT-500, Lanzhou Zhongke Kaihua Technology Development Co., Ltd., Lanzhou, China).

### 2.5. Worn Surface and Wear Residue Characterization

The wear morphologies of the worn surfaces of the balls (wear scars) and the discs (wear tracks) were examined using a JEOL JSM-7000F (JEOL Ltd., Tokyo, Japan) scanning electron microscope (SEM) with 15 kV operating voltage. Moreover, the wear tracks were characterized using a Bruker Contour GT-K (Bruker Nano, Inc., Tucson, AZ, USA) white light interferometer (WLI) with a 400–700 nm wavelength light source to acquire the three-dimensional surface topography. The wear tracks were examined using Raman spectroscopy and XPS for chemical phase analysis. The wear tracks were further treated with ultrasonic cleaning in an ethanol bath to collect MLC wear residues. These MLC wear residues were then observed with a TEM to determine changes to its morphology before and after the tribo-experiment.

## 3. Results and Discussion

### 3.1. Composition and Structural Analysis of MLC

**Figure 1:**
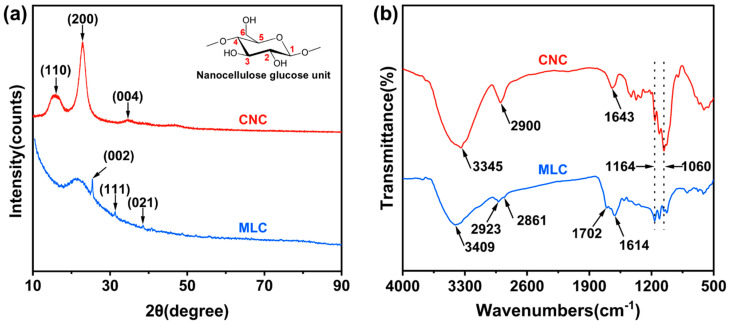
(**a**) XRD spectra and (**b**) FTIR spectra of MLC and CNC.

Figure 1a depicts the XRD spectra of MLC and CNC. The spectrum of CNC shows its characteristic peaks at 2θ values of 16.1°, 22.9°, and 34.8°, corresponding to the (110), (200), and (004) lattice planes in cellulose crystalline structure. Moreover, the Segal’s equation calculation results show that CNC has a high crystallinity of nearly 82.0% [38]. Compared with CNC, MLC shows a much different XRD spectrum, in which the characteristic peaks belonging to cellulose crystal disappear completely. However, as can be seen in the XRD spectrum of MLC, there emerges a new broad hump at 2θ of 21.3°, ascribed to amorphous carbon, and three small peaks at 2θ of 25.4°, 31.2°, and 38.5°, which are attributed to (002), (111), and (021) lattice planes of graphitized carbon, respectively. The above analysis indicates that CNC has been carbonized completely into MLC via the IL-thermal method and that MLC is primarily composed of amorphous carbon and crystalline carbon.

Figure 1b shows the FT-IR spectra of MLC and CNC. The characteristic absorption peak at 3345 cm^−1^ in the spectrum of CNC can be attributed to –OH stretching vibration [39]. Interestingly, it exhibits a blue shift of 64 cm^−1^ (to 3409 cm^−1^) in the spectrum of MLC, suggesting the presence of plenty of H-bonds between CNC powders and that H-bond interactions between MLC powders have been greatly weakened due to the carbonization. The broad peak at approximately 2900 cm^−1^ is assigned to the stretching vibration of –CH–/–CH_2_– moieties [40], as further seen from the spectrum of CNC, whereas it has been split into two peaks at 2923 cm^−1^ and 2861 cm^−1^ in the spectrum of MLC, which are assigned to –CH–/–CH_2_– symmetrical and asymmetrical stretches [41,42]. The spectrum of MLC exhibits a novel absorption peak at 1702 cm^−1^, assigned to the –C=O stretching vibration [43], which confirms the first-stage dehydration in glucose units. The absorption peak at 1643 cm^−1^ in the spectrum of CNC is mainly attributed to –OH bending vibration [44], but it has disappeared completely in that of MLC due to the dehydration reaction. The absorption peak at 1614 cm^−1^, newly emerged in the spectrum of MLC, can be ascribed to –C=C– stretching vibration [45], denoting the further stage of the dehydration reaction. Moreover, the two peaks at 1164 and 1060 cm^−1^ in both spectra are assigned to C–O–C stretching vibration and C–O stretching vibration [46,47], but their peak intensity ratio is reversed because of the carbonization. Apparently, MLC has retained a moiety of oxygen-containing groups, as compared to those common carbon nanomaterials (such as carbon nanotubes and graphene) which are inherently hydrophobic and hydrophilic [48].

**Figure 2:**
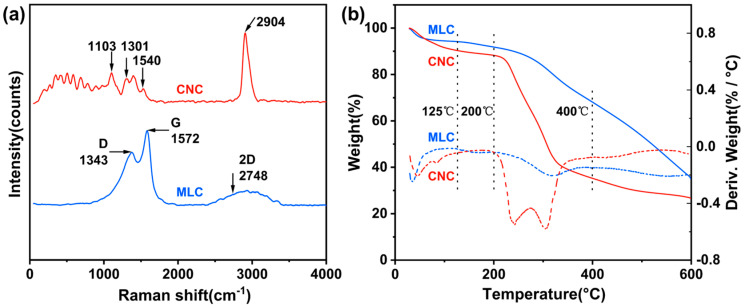
(**a**) Raman spectra, and (**b**) TGA and DTG curves of MLC and the CNC.

The Raman spectra of MLC and the CNC are presented in Figure 2a. The spectrum of the CNC exhibits a characteristic peak at 2904 cm^−1^, which can be assigned to the stretching vibrations of –CH– or –CH_2_– [49]. The other Raman peaks at 1540, 1301, and 1103 cm^−1^ can be ascribed to the shearing vibration of –CH_2_– or C–OH, the bending vibration of –CH_2_–, and the stretching vibration of C–O–C (glycosidic bond) [50,51,52]. Compared to the CNC, MLC exhibits a completely different Raman spectrum, in which the characteristic peaks corresponding to the CNC have disappeared. Moreover, two new Raman peaks appear at 1343 and 1572 cm^−1^, which correspond to the D band and the G band of amorphous carbons and graphitized carbons [53,54]. Apparently, this further confirms the XRD analysis results (see Figure 1). In addition, the new Raman peak at around 2748 cm^−1^ is attributed to the 2D band of these carbons [55], denoting MLC is most likely multi-layer stacked.

Figure 2b presents the TGA and DTG curves of MLC and the CNC. Apparently, at temperatures below 125 °C, the mass loss is ca. 5.5% for MLC and 10.0% for the CNC, corresponding to those adsorbed water molecules [56]. This indicates that the hydrophilicity of MLC has been largely reduced after carbonization. At the temperature ranging from 125 °C to 200 °C, MLC and CNCs show no significant sign of mass loss. However, the CNC has experienced a great mass loss of up to 55.1% at the temperature between 200 °C and 400 °C, which is primarily due to the thermal decomposition of glycosidic linkages and glucuronic acid moieties of anhydroglucose units [57]. In contrast, MLC has a mass loss of nearly 24.6% within the same temperature range, primarily attributed to the thermolysis of anhydroglucose residues. As temperatures exceeding 400 °C, MLC or the CNC exhibits continuous mass loss, converting into more thermally stable carbonous structures. The residual mass at 600 °C is 35.5% for MLC and 26.7% for the CNC. The above analysis indicates that MLC, as compared to the CNC, has a much higher thermostability.

**Figure 3:**
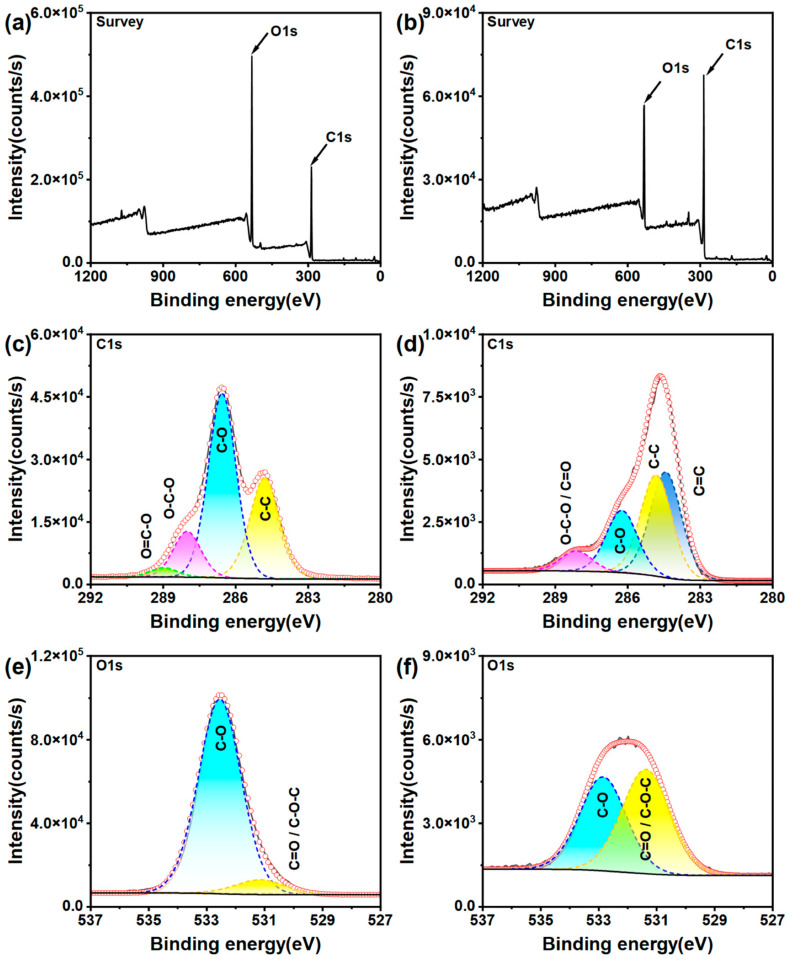
Wide scan XPS spectra, and C1s and O1s fitting spectra of (**a**,**c**,**e**) the CNC and (**b**,**d**,**f**) MLC.

The XPS wide-scan spectra of the CNC and MLC, and their corresponding C1s and O1s fitting spectra are presented in Figure 3. The atomic percentages of C and O are presented in Table 1. As seen from Figure 3a,b, the C1s and O1s peaks are clearly visible in the two wide-scan spectra, but their peak intensity ratio (C1s:O1s) becomes much higher for MLC. Moreover, C and O elements have atomic percentages of 56.1% and 43.9% in the CNC, respectively, while MLC contains 77.0% C and 23.0% O. Therefore, it can be concluded that the CNC has been subjected to serious deoxidization to be converted into MLC.

The C1s fitting spectrum of the CNC can be deconvoluted into four subpeaks at 288.98, 288.01, 286.56, and 284.8 eV and with peak-area proportions of 2.6%, 13.2%, 52.9%, and 31.3%, assigned to O–C=O, O–C–O, C–O, and C–C bonds [58,59]. The C1s spectrum of MLC splits into four subpeaks attributed to O–C–O/C=O (288.13 eV), C–O (286.17 eV), C–C (284.80 eV), and C=C (284.38 eV) bonds [60]. Compared with the CNC, MLC demonstrates a significant difference in its C1s spectral profile. Specifically, the C=C subpeak emerges with a peak-area proportion of 36.5%, while the O–C=O subpeak vanishes. Furthermore, the peak-area proportion of C–C increases to 35.0%, whereas those of C–O and O–C–O/C=O decrease significantly. This suggests that carbonizing the CNC into MLC is essentially a deoxygenation process, involving the removal of hydroxyl and carboxyl on glucose units. The O1s spectra are fitted with two peaks corresponding to C–O and C–O–C/C=O bonds for both of MLC (532.85 eV; 531.37 eV) and the CNC (532.55 eV; 531.16 eV) [61,62,63]. However, their peak-area proportions have shown a great difference for the CNC and MLC. Once the CNC was carbonized into MLC, the proportion of C–O is significantly reduced from 89.9% to 47.4% and that of C–O–C/C=O increased to 52.6% from 10.1%. It can be inferred from the above analysis that most hydroxy has been removed while inner ether and glycosidic bonds are partially retained in MLC. These changes in MLC adequately account for the above TGA results, leading to the significantly reduced mass loss below 125 °C and in the 200–400 °C range.

**Figure 4:**
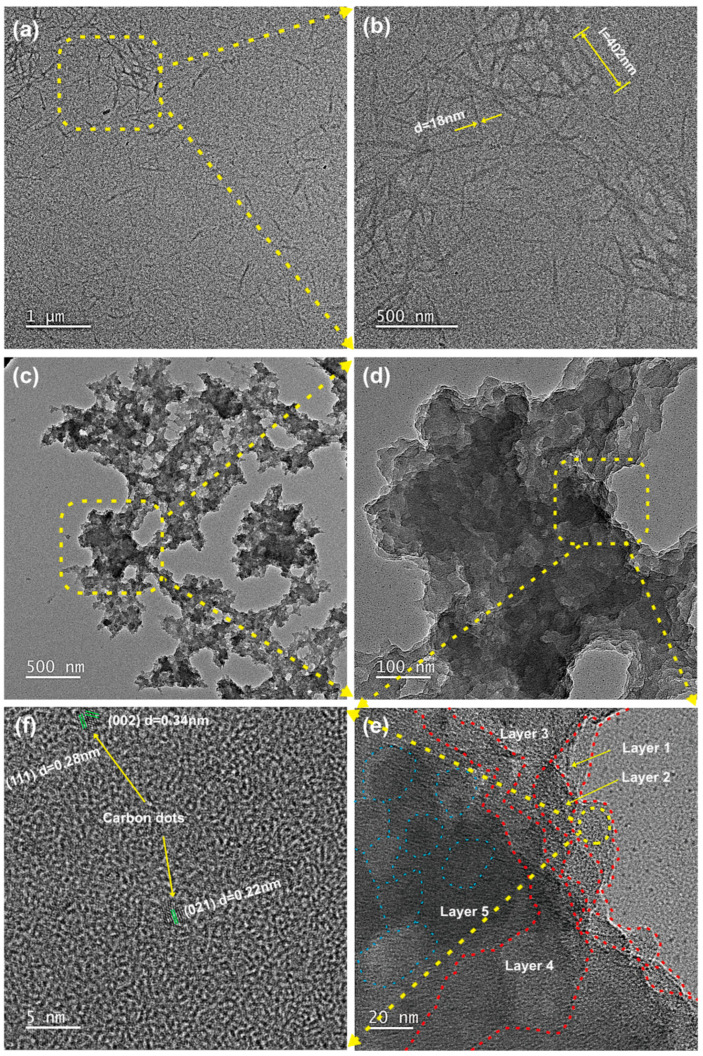
TEM micrographs showing (**a**,**b**) the CNC and (**c**–**f**) MLC.

Figure 4 presents the TEM micrographs of the CNC and MLC. As seen from Figure 4a,b, the CNC clearly exhibits a needle-like morphology with dimensions of 200–400 nm in length and 10–20 nm in diameter. Moreover, the CNC exhibits an obvious propensity to agglomerate because of interparticle H-bonds. In comparison with the CNC, MLC is still at the nanoscale but exhibits a distinctly different morphology, which can be described as a two-dimensional multilayer structure analogous to graphene, as shown in Figure 4c,d. It is apparent that MLC is formed through layer-by-layer stacking, as can be further observed in Figure 4e. Each MLC layer shows distinct, irregular edges (marked with red dotted lines), and it seems that each layer is composed of many fish-scale-like microregions (marked with blue dotted lines). More structural details are observed in the high-resolution TEM micrograph of a typical MLC layer. Apparently, extensive amorphous regions and several highly crystalline dots are contained within each MLC layer, as shown in Figure 4f. Precise measurements show that these crystalline dots have three distinct lattice spacings of 0.34, 0.28, and 0.22 nm, corresponding to (002), (111), and (021) lattice planes of crystalline carbon, which confirms the earlier XRD analysis results.

### 3.2. Dispersibility of MLC in Rapeseed Oil

**Figure 5:**
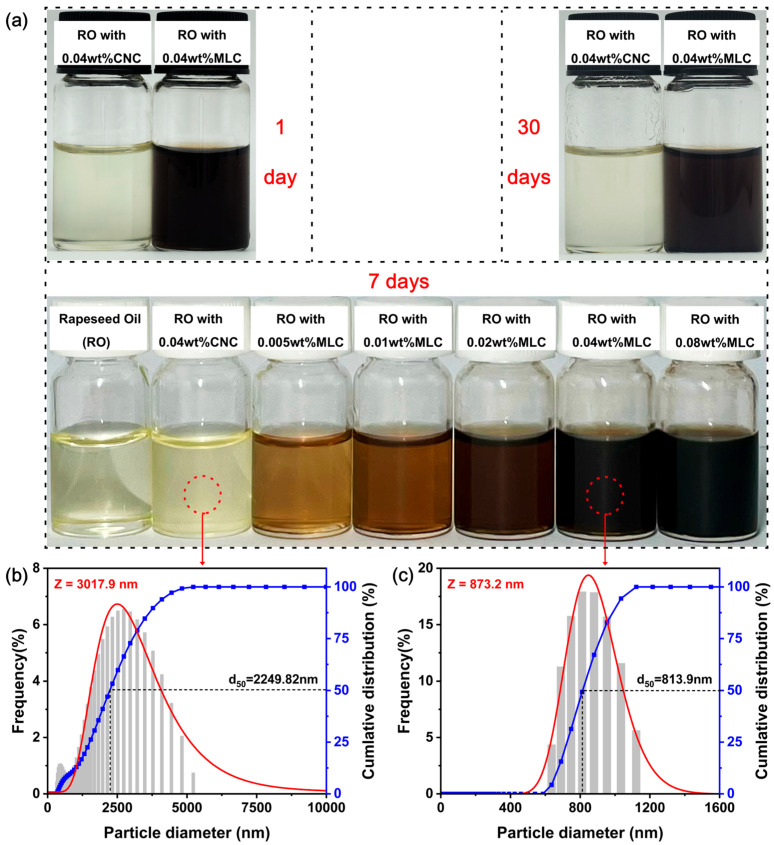
(**a**) Digital photographs of typical oil samples after standing for 1, 7, and 30 days; size distributions of particles in (**b**) RO + 0.04 wt% CNC and (**c**) RO + 0.04 wt% MLC.

The digital photographs of different oil samples (RO:rapeseed oil, RO + CNC:RO with CNC, and RO + MLC:RO with MLC), and the particle-size distributions of the CNC and MLC in rapeseed oil are shown in Figure 5. The RO + CNC and RO + MLC nano-oils, as seen from Figure 5a, remain stable and well-dispersed after 1 or 7 days of standing. Even after 30 days, no obvious phase separation or sedimentation can be observed, which indicates qualified dispersibility. Compared with graphene used in vegetable oil, which was reported to exhibit significant sedimentation after only 24 h of standing [64], MLC has superior dispersion stability. The nano-oils differ from pure RO in appearance. RO + CNC, which looks turbid, is an opaque suspension; RO + MLC appears to be translucent at a relatively low concentration but deepen its color with an increase in the MLC concentration, indicating that it is approximately a colloid. Additionally, the dispersibility is further quantitatively analyzed for RO + CNC and RO + MLC, as seen from Figure 5b,c. The particle-size distribution of CNC or MLC is approximately normally distributed. RO + 0.04 wt% CNC has a median diameter (d50) of nearly 2250 nm, but that for RO + 0.04 wt% MLC is reduced to 814 nm. The mean diameter (Z) of MLC in rapeseed oil is ca. 873 nm, much smaller than that of the CNC in rapeseed oil (3018 nm). Therefore, it can be determined that MLC has a much better dispersibility than the CNC due to its lowered surface polarity (caused by the carbonization).

### 3.3. Tribological Performance

**Figure 6:**
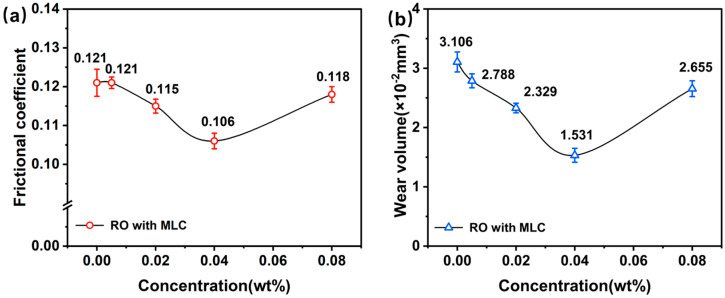
(**a**) Frictional coefficient and (**b**) wear volume plotted against MLC concentration in rapeseed oil (10 N, 200 rpm, 30 min).

Figure 6 illustrates the relationship between the MLC concentration and both the frictional coefficient and the wear volume. Clearly, the frictional coefficient decreases and then increases with increasing MLC concentration, even though the impact is limited. The frictional coefficient is still as high as 0.106 even at the MLC concentration of 0.04 wt%, which is only 12.4% less than that of pure rapeseed oil. However, the wear volume, unlike the frictional coefficient, is much more sensitive to the MLC concentration. As the MLC concentration increases, the wear volume rapidly decreases and then goes up. The wear volume has reached its minimal level at the MLC concentration of 0.04 wt%, namely 1.531 × 10^−2^ mm^3^, which is 50.7% smaller than that at zero concentration. The above analysis has shown that MLC has a comparatively stronger capability for reducing wear than reducing friction, and the optimal MLC concentration is at ca. 0.04 wt%. The anti-wear performance of MLC is comparable to that of common carbon nanomaterials, e.g., graphene. Moreover, compared with graphene, which achieves optimal performance at ~0.5 wt% [65], MLC delivers equivalent efficacy at a significantly lower concentration.

**Figure 7:**
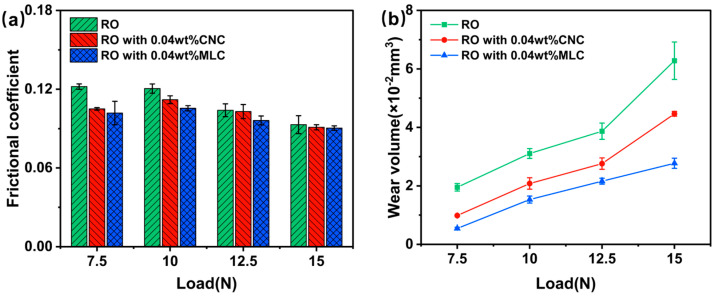
(**a**) Frictional coefficient and (**b**) wear volume vs. applied load for rapeseed oil (RO), RO + 0.04 wt% CNC, and RO + 0.04 wt% MLC. (200 rpm, 30 min).

Figure 7 presents the variations in the frictional coefficient and the wear volume with the applied load for RO, RO + 0.04 wt% CNC, and RO + 0.04 wt% MLC. As seen from Figure 7a, the frictional coefficient for RO gradually reduces as the applied load increases. RO + 0.04 wt% MLC shows the lowest frictional coefficient in the applied load range of 7.5–15 N, followed by RO + 0.04 wt% CNC, then RO. For instance, the frictional coefficients for RO + 0.04 wt% MLC, RO + 0.04 wt% CNC, and RO are 0.101, 0.105, and 0.122, respectively. This demonstrates the effectiveness of MLC in improving friction performance of RO. As seen from Figure 7b, the wear volume for each oil sample largely and persistently increases with the rise in the applied loads. Thereout, the wear volume is more influenced by the applied load than the frictional coefficient. RO + 0.04 wt% MLC always has a lower wear volume under applied loads ranging from 7.5 to 15 N, compared with RO + 0.04 wt% CNC or, especially, RO. This result indicates that MLC exhibits higher wear-reduction efficiency than the CNC.

**Figure 8:**
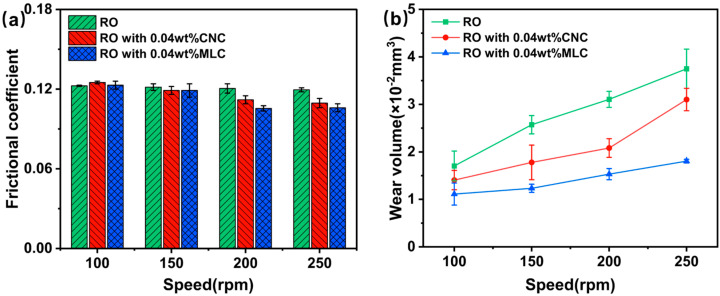
(**a**) Frictional coefficient and (**b**) wear volume vs. rotational speed for RO, RO + 0.04 wt% CNC, and RO + 0.04 wt% MLC. (10 N, 30 min).

Figure 8 illustrates the variations in the frictional coefficient and the wear volume with the rotational speed for RO, RO + 0.04 wt% CNC, and RO + 0.04 wt% MLC. The frictional coefficient, as seen from Figure 8a, is only slightly affected by the rotational speed for each oil sample. Moreover, the frictional coefficients remain at a relatively high level of ca. 0.12 at a rotational speed below 200 rpm. As the rotational speed exceeds 200 rpm, the frictional coefficient for RO shows negligible variation, but that for RO + 0.04 wt% CNC or RO + 0.04 wt% MLC starts to decrease somewhat. It can be reasonably concluded that a relatively high rotational speed is needed to realize the friction-reduction potential of the CNC or MLC. As seen in Figure 8b, similarly to the applied load, the wear volume is also highly dependent on the rotational speed for all the oil samples. The wear volume rises as the rotational speed increases, but RO + 0.04 wt% MLC consistently shows the lowest wear volume among these oil samples, demonstrating MLC’s superior antiwear performance at the appropriate rotational speeds.

**Figure 9:**
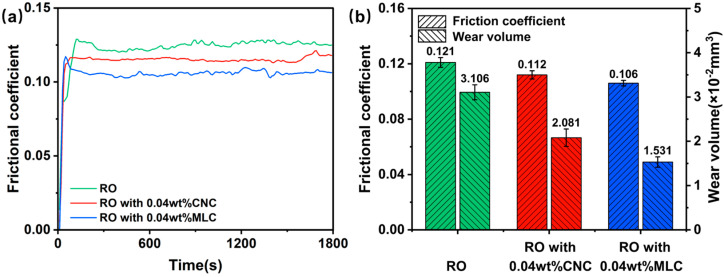
(**a**) Frictional coefficient as a function of time; (**b**) mean frictional coefficient and wear volume for RO, RO + 0.04 wt% CNC, and RO + 0.04 wt% MLC (10 N, 200 rpm, 30 min).

Figure 9 presents the frictional coefficient-time curves for RO, RO + 0.04 wt% CNC, and RO + 0.04 wt% MLC, along with the mean frictional coefficient and wear volume. Clearly, a similar variation trend can be observed for each frictional coefficient curve. The frictional coefficient increases rapidly within the initial minutes, corresponding to the run-in phase, and then remains essentially stable until the end, as shown in Figure 9a. Apparently, RO shows a longer run-in phase as compared to RO + 0.04 wt% CNC and RO + 0.04 wt% MLC. In addition, the frictional coefficient curve for RO is always higher than that for RO + 0.04 wt% CNC, which remains consistently above that for RO + 0.04 wt% MLC. This indicates that the run-in phase is effectively shortened and the friction-reducing performance is improved by using the CNC and especially MLC as nanoadditives. The mean frictional coefficients are 0.121, 0.112, and 0.106 for RO, RO + 0.04 wt% CNC, and RO + 0.04 wt% MLC, indicating a moderate improvement in the friction-reduction performance due to the CNC or MLC nanoadditive. In contrast, the anti-wear performance is improved significantly when using the CNC or MLC as nanoadditives, as seen in Figure 9b. Specifically, the mean wear volume decreases from 3.11 × 10^−2^ mm^3^ (RO) to 2.08 × 10^−2^ mm^3^ (RO + 0.04 wt% MLC) or 1.53 × 10^−2^ mm^3^ (RO + 0.04 wt% CNC). Overall, while their friction-reducing effects are not outstanding, both the CNC and MLC show considerable efficacy in wear reduction, with MLC consistently outperforming the CNC.

### 3.4. Wear Analysis

**Figure 10:**
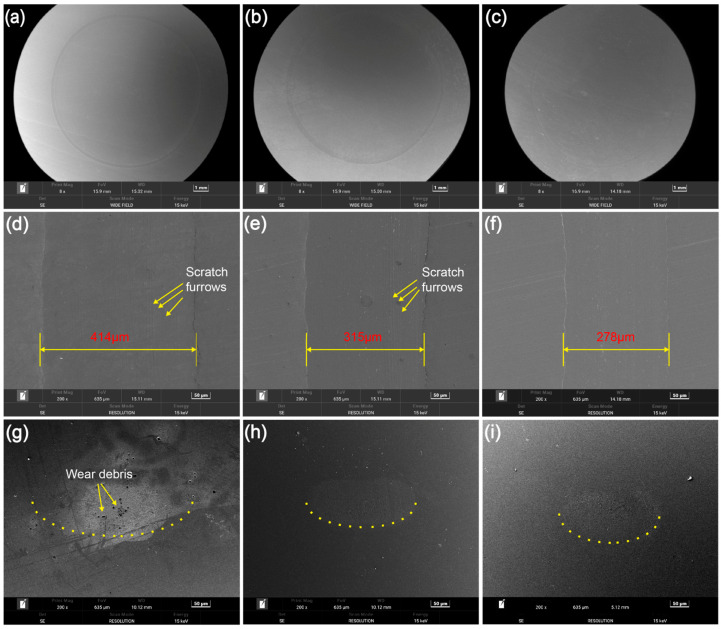
SEM micrographs of worn surfaces on (**a**–**f**) steel discs and on (**g**–**i**) steel balls under lubrication of (**a**,**d**,**g**) RO, (**b**,**e**,**h**) RO + 0.04 wt% CNC, and (**c**,**f**,**i**) RO + 0.04 wt% MLC (10 N, 200 rpm, 30 min).

Figure 10 shows the SEM micrographs of worn surfaces of the balls (wear scars) and the discs (wear tracks) lubricated with different oil samples. All the wear tracks manifest as annular patterns with a uniform width and a radius of 5 mm, as can be seen in Figure 10a–c. However, their localized enlarged views reveal distinct variations in their width, as seen in Figure 10d–f. The wear track associated with RO has a width of nearly 414 μm, that associated with RO + 0.04 wt% CNC has its width reduced to 315 μm, and that associated with RO + 0.04 wt% MLC shows a lower width of merely 278 μm compared to the preceding two. In addition, the wear track associated with RO + 0.04 wt% MLC shows a much smoother worn surface than the other two wear tracks, which exhibit some furrows and scratches on their surfaces, indicating the occurrence of abrasive wear [66]. More interestingly, as shown in Figure 10g, some wear debris are adhered to the worn surface on the ball lubricated with RO, which indicates adhesive wear has occurred [67], whereas the worn surface on the ball lubricated with RO + 0.04 wt% CNC or RO + 0.04 wt% MLC is approximately an elliptical wear scar without any wear debris, as shown in Figure 10h,i. Based on the above analysis, it can be concluded that the friction pair under lubrication of RO suffers from severe abrasive wear and adhesive wear, but only moderate abrasive wear occurs after adding CNC as a nanoadditive, and mild abrasive results from using the MLC nanoadditive.

**Figure 11:**
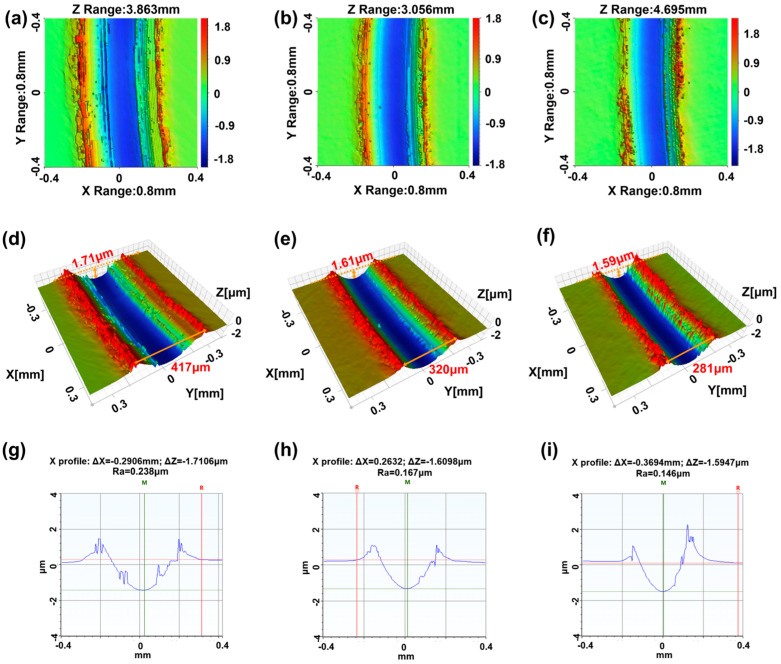
(**a**–**f**) WLI images and (**g**–**i**) across-sectional profiles of wear tracks on the discs lubricated with (**a**,**d**,**g**) RO, (**b**,**e**,**h**) RO + 0.04 wt% CNC, and (**c**,**f**,**i**) RO + 0.04 wt% MLC (10 N, 200 rpm, 30 min).

Figure 11 shows the WLI images and the across-sectional profiles of the wear tracks on steel discs lubricated with three distinct oil samples to analyze their wear morphology in more detail. The wear tracks are marked with red on both lateral sides and blue at their center, as shown in Figure 11a–c, indicating two ridges and a valley along the sliding direction. Apparently, the plastic deformation has occurred on the disc due to the squeezing effect of the ball. These analysis results are clearly visible in the 3D topography images of the three wear tracks, as shown in Figure 11d–f. Moreover, the maximum depth beneath the steel disc’s base surface (Dmax) and the wear track width can also be observed for each wear track. The Dmax of the wear track associated with RO (rapeseed oil), RO + 0.04 wt% CNC, and RO + 0.04 wt% MLC are 1.71, 1.61, and 1.59 μm, respectively. The wear track widths associated with RO, RO + 0.04 wt% CNC, and RO + 0.04 wt% MLC are 417, 320, and 281 μm, which match the measured values in Figure 10d–f. The surface roughness (Ra) of the wear tracks was measured based on their typical across-sectional profiles, as seen in Figure 11g–i. The Ra values for RO, RO + 0.04 wt% CNC, and RO + 0.04 wt% MLC were 0.238, 0.167, and 0.146 μm, respectively. Therefore, a visual observation shows that the RO + 0.04 wt% MLC can result in a smaller wear volume and a smoother wear track than both RO + 0.04 wt% CNC and RO.

**Figure 12:**
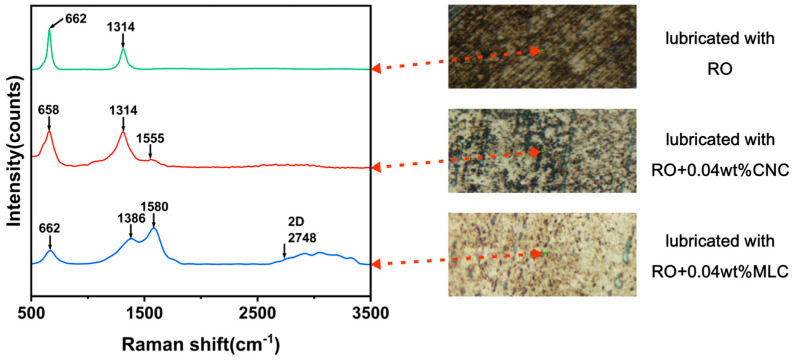
Raman spectra on wear tracks under lubrication of RO, RO + 0.04 wt% CNC, and RO + 0.04 wt% MLC. (10 N, 200 rpm, 30 min).

The Raman spectra on the wear track surface of steel discs lubricated with three different oil samples are presented in Figure 12 to further examine wear residues. The characteristic peak at 662 cm^−1^ can be ascribed to iron oxides [68], indicating the freshly exposed steel tribopair interface continues to oxidize under lubrication of RO, as seen in the Raman spectrum corresponding to RO. Moreover, that at 1314 cm^−1^ is attributable to a number of rapeseed oil molecules remaining on the worn surface [69]. In addition to the characteristic peak of iron oxides (658 cm^−1^) and that of a few rapeseed oil molecules (1314 cm^−1^), as shown in the Raman spectrum corresponding to RO + 0.04 wt% CNC, a new but weak peak appears at 1555 cm^−1^, corresponding to CNC residues [50], which indicates that CNC nanoadditives have participated in the tribological process. The Raman spectrum corresponding to RO + 0.04 wt% MLC not only shows the Raman peak of iron oxide (662 cm^−1^), but exhibits the Raman peaks at 1386 cm^−1^ (D band) and 1580 cm^−1^ (G band), which is due to the presence of MLC residues. This suggests that MLC tends to adhere to the steel tribopair interface and actively participates in the tribological process.

**Figure 13:**
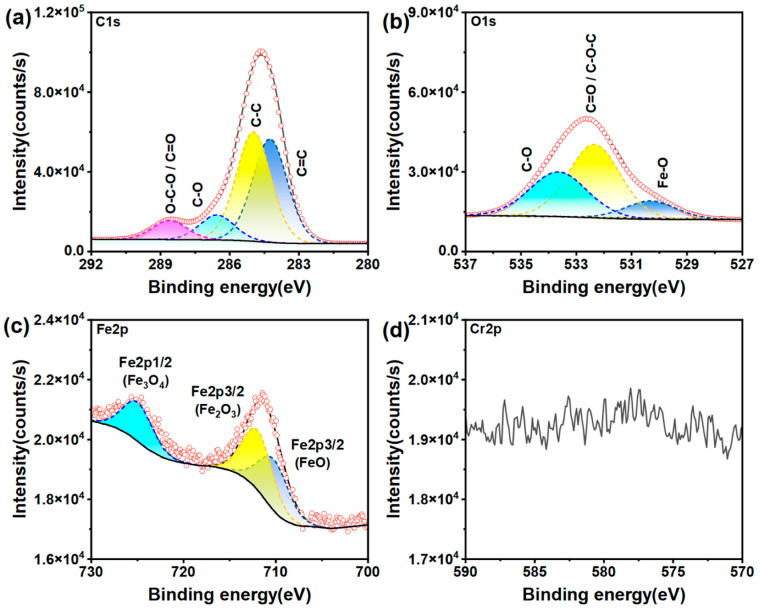
(**a**) C1s, (**b**) O1s, (**c**) Fe2p, and (**d**) Cr2p fitting XPS spectra for the wear track on the steel disc lubricated with RO + 0.04 wt% MLC (10 N, 200 rpm, 30 min).

Figure 13 depicts C1s, O1s, Fe2p, and Cr2p fitting XPS spectra for the wear track on the steel disc lubricated with RO + 0.04 wt% MLC. The C1s spectrum, as shown in Figure 13a, is deconvoluted into four subpeaks assigned to C=C (284.32 eV), C–C (284.80 eV), C–O (286.31 eV), and C–O–C/C=O (288.46 eV) bonds, respectively [60]. The distinct C=C subpeak demonstrates the presence of MLC nanoadditives on the wear track. The O1s spectrum, as shown in Figure 13b, can be fitted with three subpeaks at 529.92 eV, 532.31 eV, and 533.57 eV, representing Fe–O, C=O/C–O–C, and C–O bonds [70]. The Fe2p spectrum splits into three subpeaks corresponding to FeO (710.21 eV), Fe_2_O_3_ (711.97 eV), and Fe_3_O_4_ (724.74 eV) [71]. This suggests that a friction-induced oxidation reaction has occurred on the wear track. However, no distinct signal was detected in the Cr2p spectrum, indicating the absence of Cr. In addition, the atomic percentage of C1s is as high as 82.26%, that of O1s is 17.01%, but Fe2p is present at only 0.31%. It can be reasonably inferred from the above analysis results that those MLC nanoadditives present on the wear track have formed a protective film, resulting in the detection of only trace amounts of Fe but no Cr.

**Figure 14:**
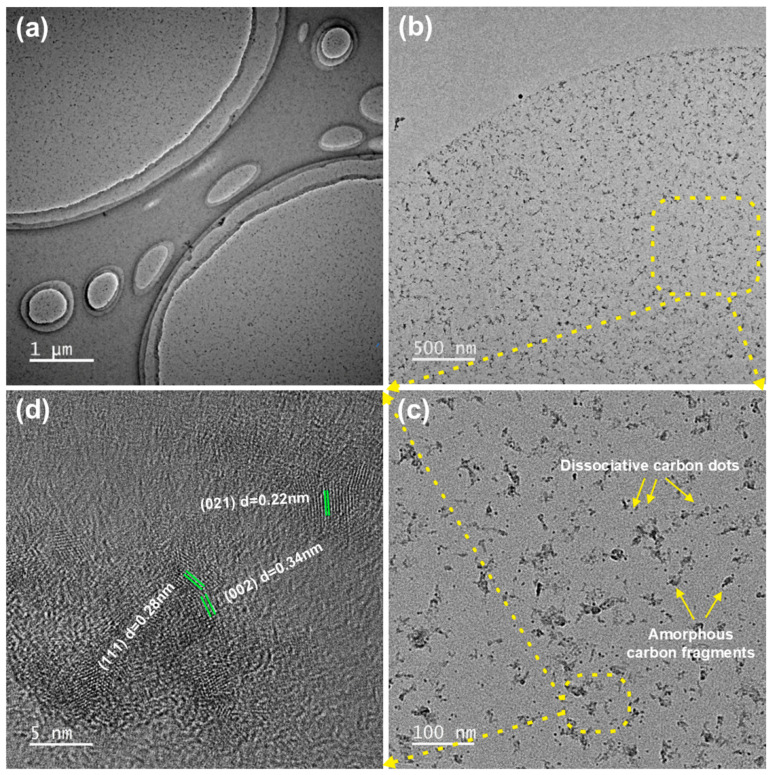
TEM micrographs of wear residues on the worn surface under lubrication of RO + 0.04 wt% MLC. (**a**) Overview; (**b**) Enlarged view; (**c**) Detailed morphology; (**d**) Lattice fringes.

The TEM micrographs of wear residues are presented in Figure 14 to further determine the friction and wear behavior of MLC. The wear residues are uniformly distributed and irregularly shaped fine fragments, as shown in Figure 14a,b, which indicates that the multi-layer structure of MLC has been severely damaged due to frictional force between the tribopair interface. More precisely, MLC wear residues are primarily composed of many dissociative carbon dots and amorphous carbon fragments, as further illustrated in Figure 14c. These carbon dots are highly crystallized with an extremely uniform size of less than 5 nm. Three types of lattice planes, namely (002), (111), and (021), can be clearly distinguished from the high-resolution TEM micrograph, as shown in Figure 14d. The above analysis suggests that copious crystalline carbon dots are released from MLC layers to further resist friction and wear, after MLC is ground into fine fragments during the tribological process.

### 3.5. Lubrication Mechanism of MLC Nanoadditive

**Figure 15:**
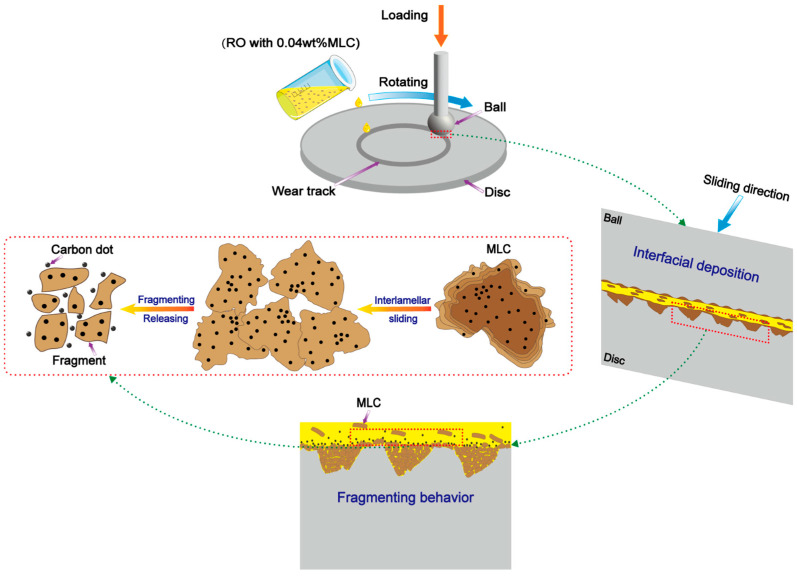
Schematic illustration of lubrication mechanism of MLC nanoadditive in rapeseed oil.

The possible lubrication mechanism of the MLC nanoadditive, which is elucidated through comprehensive analysis of both the worn surfaces and the wear residues, is illustrated in Figure 15. Under the lubrication of RO + MLC nano-oil, numerous MLC nanoadditives can be effortlessly entrained into the tribological contact zone between the tribopair together with RO molecules, due to their excellent dispersibility in RO (Figure 5’s result). The entrained MLC is prone to depositing on the tribopair interface through adsorption (Figure 12 and Figure 14’s results), and thus, protective deposit films are formed on the interface (Figure 13’s result). On the one hand, the deposit films not only significantly reduce direct contact between the tribopair interface [72], but also distribute the applied load and withstand the shear forces. This, therefore, leads to a major shift in wear mode (Figure 10 and Figure 11’s results) and a reduction in friction and, especially, wear (Figure 6, Figure 7 and Figure 8’s results). On the other hand, those MLCs, constituting the protective deposit film, are inevitably subjected from continuous friction force and friction heat. Due to its unique multi-layer stacking nanostructure (Figure 4’s result), MLC should be forced to undergo interlayer slippage under shear stress, which largely reduces the local stress concentration. Moreover, MLC nanosheets are prone to fracturing into numerous small fragments due to the friction force, and thus, copious carbon dots are released from MLC layers (Figure 14’s result). This fragmenting behavior is beneficial for dissipating the frictional force. These released carbon dots could also induce a rolling effect on the tribopair interface to further reduce friction and wear [73].

## 4. Conclusions

Overall, a novel MLC was synthesized by carbonizing the CNC with a newly developed ionic-liquid thermal method at ambient pressure and 150 °C. During carbonization, the CNC was converted into MLC via dehydration reactions. MLC was primarily composed of amorphous carbons and crystalline carbon dots, with a high carbon content of 77.0 at%. MLC showed a unique two-dimensional multi-layered microstructure and morphology. In each MLC layer, copious highly-crystalline carbon dots were embedded in the amorphous carbon nanosheet. MLC demonstrated excellent dispersibility and stability when dispersed in rapeseed oil for use as a lubricant nanoadditive. The tribological experiments showed that the performance of MLC was highly dependent on its concentration, applied load, and rotational speed; MLC was more effective at reducing wear than at reducing friction. Under appropriate experimental conditions (0.04 wt%, 10 N, 200 rpm, and 30 min), MLC induced a decline in the frictional coefficient by 12.4% and the wear volume by 50.7% compared to rapeseed oil, outperforming the CNC nanoadditive. Additionally, the wear analysis results revealed that the lubrication mechanism of the MLC nanoadditive could be attributed to its adsorptive deposition behavior and fragmenting behavior on the tribopair interface. Direct contact between the tribopair interface could be greatly reduced by MLC after deposition. Meanwhile, the frictional force was dissipated as MLC fractured and numerous crystalline carbon dots released to further induce a rolling effect. Our findings establish a novel approach for preparing nanosized biocarbon and highlight its promising potential in lubrication applications.

## Figures and Tables

**Table 1:** C and O atomic percentages in the CNC versus MLC.

Sample	Atomic Percentage (%)	
	C	O
CNC	56.1	43.9
MLC	77.0	23.0

## Data Availability

The original contributions presented in this study are included in the article. Further inquiries can be directed to the corresponding author.
